# Co-creation of a training for community health workers to enhance skills in serving LGBTQIA+ communities

**DOI:** 10.3389/fpubh.2023.1046563

**Published:** 2023-03-16

**Authors:** Vanessa Kitzie, Julie Smithwick, Carmen Blanco, M. Greg Green, Sarah Covington-Kolb

**Affiliations:** ^1^School of Information Science, University of South Carolina, Columbia, SC, United States; ^2^Center for Community Health Alignment, University of South Carolina Arnold School of Public Health, Columbia, SC, United States

**Keywords:** LGBTQ, LGBTQIA+, community health workers, CHW training protocol, curriculum development

## Abstract

This paper describes creating and implementing a 30-h LGBTQIA+ specialty training for community health workers (CHWs). The training was co-developed by CHW training facilitators (themselves CHWs), researchers with expertise in LGBTQIA+ populations and health information, and a cohort of 11 LGBTQIA+ CHWs who theater tested and piloted the course. The research and training team collected cohort feedback through focus groups and an evaluative survey. Findings stress the importance of a curriculum designed to elicit lived experiences and informed by a pedagogical framework centered on achieving LGBTQIA+ visibilities. This training is a vital tool for CHWs to foster cultural humility for LGBTQIA+ populations and identify opportunities to support their health promotion, especially considering their limited and sometimes absent access to affirming and preventative healthcare. Future directions include revising the training content based on cohort feedback and adapting it to other contexts, such as cultural humility training for medical and nursing professionals and staff.

## 1. Introduction

LGBTQIA+ populations experience significant health and healthcare disparities compared to their heterosexual, cisgender (i.e., people whose gender identities align with their sex assigned at birth) peers (1–3). These disparities arise in part because LGBTQIA+ people lack access to health-protective resources, including financial resources, affirming healthcare (i.e., healthcare that supports people's sexualities and gender identities), and social safety (4–7). Considering these disparities, LGBTQIA+ populations exhibit resilience when promoting individual and community health. Examples of resilient practices are developing positive coping strategies, resisting stigma and discrimination, and producing and exchanging new forms of affirming health information (8–10). One promising avenue to support these practices is training LGBTQIA+ leaders as community health workers (CHWs).

A CHW is a “frontline public health worker who is a trusted member of and/or has an unusually close understanding of the community served. This trusting relationship enables the worker to serve as a liaison/link/intermediary between health/social services and the community to facilitate access to services and improve the quality and cultural competence of service delivery” (11). In their study of the health information practices of LGBTQIA+ people and communities in South Carolina (SC), Kitzie et al. (9) identified community leaders who informally served in CHW roles. Informed by these findings, the research team, in partnership with the Center for Community Health Alignment (CCHA) at the Arnold School of Public Health, recruited 11 SC LGBTQIA+ community leaders and trained them to become CHWs. As part of this training, the team partnered CHWs with academic librarians to co-create informational resources for the CHWs' communities. This paper reports on one project element: co-developing an LGBTQIA+ specialty training to build on CHW training and skills.

To the best of the authors' knowledge, this specialty training represents one of the first in the US to center LGBTQIA+ populations explicitly. It is intended for all CHW audiences (LGBTQIA+ and non-LGBTQIA+) and will develop and grow their cultural humility for LGBTQIA+ people and ability to identify opportunities for supporting their health promotion. This paper will outline how the team developed the training, lessons learned from cohort feedback, and future directions. Evidence-based implications can inform the development of similar trainings.

## 2. Background and rationale for the educational activity innovation

### 2.1. Background

#### 2.1.1. CHWs and core competency training

CHWs work in communities that lack access to affirming and preventative healthcare. It is critical that CHWs share lived experiences with these communities (12). CHWs help communities access resources for their health needs, deliver professional development trainings to providers to increase their cultural humility, address mistrust of healthcare institutions by serving as a trusted bridge or cultural liaison, and engage in advocacy work to improve healthcare resources for critically underserved populations (12). A growing body of research has found associations between CHWs and improved health outcomes, utilization of appropriate healthcare services, and service cost-effectiveness (13–15).

The C3 Project, a national consensus-driven process to enhance cohesion around the CHW model of care, established a set of CHW roles and competencies that have become the basis for CHW training and practice in SC and nationwide (11, 12). The SC CHW Credentialing Council approves the CHW core competency training. It requires curricula to cover the skills identified by the C3 Project, along with two additional competencies related to quality of care and health equity.

CCHA offers a 160-h CHW core competency training. The training includes 2.5 h of content that guides culturally humble care to LGBTQIA+ communities. A variety of case studies throughout the core competency training also ask learners to consider how to work with participants who have diverse gender identities and sexualities.

#### 2.1.2. LGBTQIA+ populations

As mentioned, LGBTQIA+ populations experience health and healthcare disparities, such as the increased risk of obesity and chronic illness, higher HIV infections and STI rates, and enhanced mental distress (16–19). Disparities vary based on identities within the LGBTQIA+ umbrella and cross-cutting identities like race and class. Pervasive stigma and discrimination against LGBTQIA+ people create insufficient social safety nets, producing these disparities (5). For instance, healthcare provides an inadequate social safety net for LGBTQIA+ populations, who often perceive doctors and hospitals as unsafe due to provider lack of knowledge, negative experiences, inability to pay for care, and provider refusal to give care (20, 21). A socio-ecological framework, which addresses health prevention efforts across four levels (societal, community, relational, and individual), offers a promising approach for identifying gaps in the social safety net spanning multiple domains, including family, peers, community, and school (22, 23).

CHWs are uniquely positioned to address gaps in LGBTQIA+ persons' social safety nets due to their relationships of trust with the people they serve and their emphasis on interventions following the socio-ecological framework (24). However, CHWs may experience similar gaps in knowledge about LGBTQIA+ experiences, identities, and needs as those experienced by medical and nursing providers and staff. Studies examining medical and nursing curricula show a median of 5 h, out of a 4-year program, devoted to LGBTQIA+ curricula-specific content hours for undergraduate medical students (25, 26) and report that 80% of nurses surveyed in 2015 did not receive any LGBTQIA+ specific training (27). Only providing information about LGBTQIA+ identities, experiences, and needs is not enough. As Stroumsa et al. (28) demonstrate, transphobia poses a significant barrier to provider knowledge about trans-specific healthcare despite the presence or absence of specific training on this topic. The effectiveness of LGBTQIA+ healthcare education initiatives “may depend not only on increasing informational knowledge but also on addressing providers' biases, whether conscious or unconscious. Educational initiatives will need to take learners' backgrounds into account, directly address prejudice and enhance cultural humility” (28).

### 2.2. Rationale

CHWs are poised to respond to barriers faced by communities experiencing marginalization. While LGBTQIA+ populations constitute one such community, their needs might not be met fully if CHW training reflects similar medical and nursing education gaps. For many CCHA trainees, the core competency training is the first time they have had open discussions about gender identity and sexuality, the importance of affirming care, and health disparities affecting LGBTQIA+ populations. Such limited exposure can further perpetuate misinformation, stigmatizing language, and other unintentional offenses toward LGBTQIA+ people. CCHA observed that more time and information than what 2.5 h of core competency training can cover is required for CHWs to establish cultural humility centered on LGBTQIA+ health promotion.

To address this need, a team of CHW training facilitators (who are CHWs) at CCHA, researchers with expertise in LGBTQIA+ populations and health information, and a cohort of 11 LGBTQIA+ CHWs co-developed a 30-h LGBTQIA+ specialty training. This training allows CHWs to take an in-depth look at specific disparities and inequities experienced by LGBTQIA+ populations at all four socio-ecological framework levels. The training focuses on fostering cultural humility. Cultural humility shifts from the mastery perspective adopted in cultural competency approaches to developing personal strategies for accountability in acknowledging power differentials between CHWs and clients and challenging the social and structural barriers to LGBTQIA+ health promotion (29).

## 3. Pedagogical frameworks

### 3.1. Popular education

Popular education is a form of adult education that emphasizes participation and encourages learners to reflect on their personal experiences to think critically about social issues. This type of education is “popular” because it is “of the people” and is a collaboration amongst all learners and facilitators who teach and learn from each other.

According to Wiggins (30), popular education “draws out and validates what participants already know and do, connects their personal experience to larger social realities, and then supports participants to work collectively to change their reality.” Popular education places value and grounds learning on the participants' experiences and knowledge, modeling the C3 Project role of “Building Individual and Community Capacity” (12). This strategy is vital because the most important quality of a CHW, according to the C3 Project, is the “connection with the community served” (12). In literature, popular education has improved participant empowerment and health outcomes (30).

### 3.2. Queer pedagogy

Queer pedagogy is “a radical form of educative praxis implemented deliberately to interfere with, to intervene in, the production of 'normalcy' in schooled subjects” (31). It challenges what educators take for granted in teaching settings, such as the banking model of education, which envisions the instructor as the only person in the room possessing knowledge (32). Rather than considering knowledge as something to be mastered, queer pedagogy instead asks questions about knowledge, such as: “Who gets to know? Who gets to be considered knowledgeable? What do we refuse to know and why?” Queer pedagogy focuses less on presenting informational knowledge about LGBTQIA+ experiences, identities, and issues and instead questions why others consider these experiences, identities, and issues to be not “normal.” Queer pedagogy encourages instructors and students alike to unlearn traditional assumptions they might have about LGBTQIA+ people based on what they take for granted as normal within systems like healthcare (33).

### 3.3. Cultural humility

Cultural humility is “a foundational concept and skill for guiding the work of CHWs” (34). Cultural humility challenges and readdresses power imbalances between the service provider and client; these imbalances have sustained discriminatory practices and contributed to inequitable access to care. Cultural humility asks learners to acknowledge the limits of their knowledge about other cultures and that cross-cultural work involves lifelong learning and self-reflection. For CHWs, this critical component of client-centered care requires openness and humility when working with individuals and defined populations. Facilitating with a culturally humble lens is imperative and fosters a safe learning environment inclusive of all diverse values, backgrounds, and identities present.

The pilot cohort and facilitators of this training represented diverse, intersectional identities (i.e., how people's identities can overlap in ways that compound the privilege/oppression they experience), including age group, ethnicity, cultural background, and LGBTQIA+ identities. Regarding critical issues discussed in this course, learners considered existing cis/heteronormative norms and how these can exacerbate health disparities within LGBTQIA+ communities. Using a culturally humble lens when working with LGBTQIA+ communities builds trust and openness. It also acknowledges that no one identity or cultural value is more meaningful or superior to others and that an individual's reality should be recognized as different from the realities of those identifying outside of cis/heteronormative norms.

## 4. Curricular outline and learning environment

### 4.1. Curricular outline

The team developed a 30-h LGBTQIA+ specialty training meant to be taken after completing the 160-h core competency training. From May–July 2021, the team began planning the curricular outline by identifying five main areas of focus, which ultimately became the course modules: (1) terminology and history of LGBTQIA+ identities (5 h); (2) intersectionality and LGBTQIA+ identities (10 h); (3) LGBTQIA+ health issues (10.5 h); (4) resources and strategies for LGBTQIA+ health promotion (2.5 h); (5) advocacy and outreach to LGBTQIA+ people and communities (2 h). The team identified these areas based on several factors, including CCHA's observations of prior CHW training participants' reception of and feedback about the 2.5 content hours focused on LGBTQIA+ topics; the team's previous research concerning health issues faced by LGBTQIA+ populations and their health information work (9, 35–37); feedback from an eight-person advisory board comprised of LGBTQIA+ community leaders, CHWs, and researchers in Public Health and Information Science fields.

The training is unique to CHWs because it focuses on the critical roles that CHWs play as information and resource intermediaries between communities experiencing marginalization and healthcare institutions (11). For instance, CHWs attending this training discuss safe housing considerations when making referrals, share pronouns when facilitating groups, and understand the need to add LGBTQIA+ affirming providers to their resources; all these activities address socio-ecological health interventions. Another reason the training is unique is that CHWs and allies developed it utilizing best practices in CHW training. Some team members who developed the training have backgrounds in Information Science, which focuses on identifying, evaluating, and disseminating health information resources; their expertise also informed the training and focused on CHW intermediary roles.

After identifying the modules, the team created specific topics and learning outcomes for each, informed by feedback from the sources above. The team also developed two sample units. The pedagogical framework informed the development of content, especially queer pedagogical approaches. Popular approaches include (1) recognizing the limits of dominant ways of knowing; (2) examining ignorance from the perspective of active resistance to learning about specific topics; (3) ensuring learning materials represent queer perspectives and experiences; (4) interrogating disclosure, specifically who must disclose what about themselves in given situations or circumstances (38). Examples of how these approaches informed content development were (1) locating LGBTQIA+ health disparities and challenges within healthcare systems and institutions—not individuals; (2) engaging in reflexive exercises meant to foster cultural humility by asking participants to identify their preexisting biases toward specific identities and issues; (3) integrating content created by LGBTQIA+ individuals with formal expertise and lived experiences into the training; (4) actively discussing labels and terminology, including using terms that actively make visible identities often not labeled or taken for granted because society considers them normal (e.g., allosexual, which refers to people who experience sexual attraction). These strategies also corresponded with popular education and cultural humility principles.

One unique element of the training's pedagogical format was the inclusion of expert videos. Before the training, the team contacted nine LGBTQIA+ people with lived experience and formal expertise in curricular subject areas and asked them to record an informational 10–15-min video presentation. An example was an academic researcher specializing in health and aging among queer, transgender, and intersex populations. The expert presented findings from their research about intersex affirmation in health care settings and reflected on these findings based on their experiences as an intersex person. The team identified experts based on their networks within the fields of Information Science and Public Health and provided each expert with $250 honoraria for their contributions.

Table 1 displays a sample unit for each module with accompanying learning objectives, definitions, and activities.

**Table 1:** Sample unit with learning objectives, definitions, and activities.

**Module 1 sample unit: LGBTQIA+ 101**
*Learning objectives:*
• Summarize LGBTQIA+ demographics in the United States
• Define basic LGBTQIA+ terms and concepts
• Explain how sex, sexuality, and gender are socially and medically constructed
• Employ LGBTQIA+ inclusive language
*Key definitions*
Gender identity; sex assigned at birth; gender expression; gender roles; sexual orientation; romantic/emotional orientation; bisexual; pansexual; asexual; transgender; cisgender; agender; genderfluid; genderqueer
*Sample activity*
In groups of 2–3, come up with a demographic question or questions you want to know about LGBTQIA+ people in SC. See if you can answer the question you came up with by searching online.
• What was your question?
• What, if anything, was challenging or difficult about this exercise?
• What do you think made this exercise challenging/difficult?
• What questions remain for you after this exercise?
**Module 2 sample unit: Introduction to intersectionality**
*Learning objectives:*
• Define intersectionality
• Describe how intersectionality originated (Truth, Crenshaw, Hill Collins)
• Dispel common intersectionality and myths
• Apply lens of intersectionality to LGBTQIA+ identities and issues
*Key definitions*
Intersectionality; matrix of domination; top-down vs. bottom-up approaches; DeGraffenreid v. General Motors
*Sample activity*
Pick a health topic you are passionate about. Write this topic on the Jamboard as well as your answers to the following questions:
• How do LGBTQIA+ people and communities experience your chosen topic?
• What other important identities might inform how LGBTQIA+ people experience this topic?
• Are there groups left out of the discussion?
• Are some groups overrepresented? Why might this be?
You may complete outside research to answer these questions.
**Module 3 sample unit: Pursuing gender affirmation**
*Learning objectives:*
• Define gender affirmation
• Provide examples of social, psychological, legal, and medical approaches to achieve gender affirmation
• Describe HRT and who uses HRT within LGBTQIA+ communities
• Identify barriers to accessing HRT within among LGBTQIA+ communities
• Develop advocacy strategies for HRT use within LGBTQIA+ communities
*Key definitions*
Gender affirmation (legal, medical, social); HRT (estrogen, testosterone, low dose); puberty blockers
*Sample activity*
In groups of 2–3 discuss:
• Do you (or someone you know) have any experience with taking hormones? Do we notice patterns in who takes hormones of their experiences? If so, what are they?
• If you do not know someone personally, see if you can find someone's account of going through HRT. What was the process they had to go through? How long did the process take? Were there any challenges or barriers they experienced?
**Module 4 sample unit: Resources for LGBTQIA**+ **health promotion**
*Learning objectives:*
• Describe insurance coverage for LGBTQIA+ people in SC
• Identify local health resources and programs that offset insurance costs for LGBTQIA+ populations
*Key definitions*
Affordable Care Act; Medicaid; health insurance marketplace; quality of life resources
*Sample activity*
Your friend is taking Fenoglide for their cholesterol. They're struggling to afford their medication and ask you if know of any pharmacies that have the generic version of their medication, fenofibrate, on their formulary list.
In breakout groups, locate a pharmacy that includes fenofibrate on their formulary list. Then, discuss:
• What was this experience like?
• What challenges may people face when seeking ways to offset medication costs?
**Module 5 sample unit: Advocacy and outreach to LGBTQIA**+ **people and communities**
*Learning objectives:*
• Ask questions about local LGBTQIA+ organizations and communities in a safe environment
• Identify tactics used by local LGBTQIA+ communities and organizations to advocate for their members
*Key definitions*
Advocacy; outreach
*Sample activity*
Pair and Share: Recall a time when you or someone you know advocated on behalf of a person or a group of people.
• What happened?
• Who was involved?
• What was the issue or issues that inspired the act of advocacy?
• Was this act effective? Why or why not?

### 4.2. Learning environment

The specialty training was online using Zoom, required participant cameras to be always on (a requirement of the credentialing body), and emphasized participatory learning. Facilitators facilitated open discussion amongst learners, reinforcing critical concepts by utilizing their life experiences and expertise. The training used learning aids, tools, and programs to foster and maintain the integrity of the participatory learning environment. Each class integrated multiple methods, including case studies and role plays using Zoom breakout groups, collaborative notetaking using Jamboard (interactive whiteboard), and knowledge checks using the game-based learning platform Kahoot!.

The facilitators covered the 30-h curriculum over 2 weeks, meeting with training participants for three consecutive hours on weekdays. Each training session began with an icebreaker and an overview of the plan for the day. Facilitators would then cover course material consisting of Google slides and multimedia, including audio clips and videos.

Following the presentation of course content, facilitators would ask training participants to engage in collaborative discussions and activities. A break followed this engagement, and the structure would resume until the session's conclusion. Facilitators provided training participants with a link to the slides and additional resources after each session.

## 5. Results to date and assessment

### 5.1. Processes and tools

**Figure 1:**
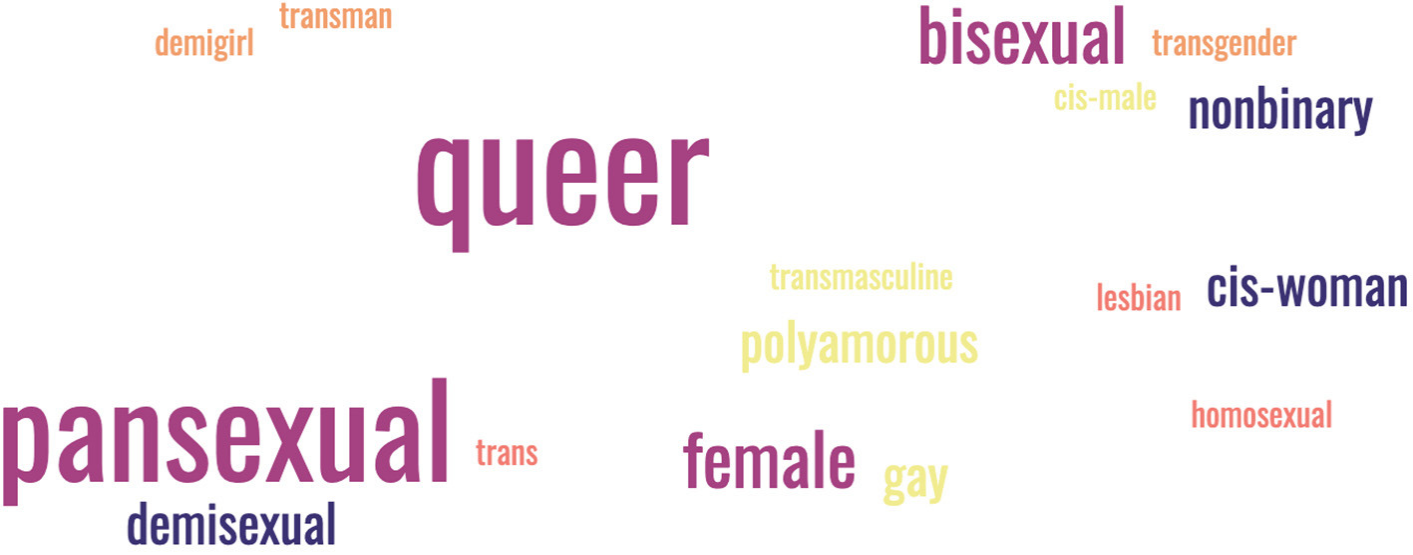
Labels used by participants to describe their sexualities and gender identities.

Receiving feedback on the training from CHWs and LGBTQIA+ people was vital. The team recruited a cohort of 11 LGBTQIA+ community leaders from SC to provide this feedback. The cohort provided input on the curriculum as it was being developed and again at the culmination of the training. Recruitment methods relied on the research team's pre-established network of participants and a contact list of visible LGBTQIA+ and affirming communities in the state. Those interested attended an interest meeting and completed a questionnaire developed by CCHA and informed by C3 standards. The team met to evaluate the responses, looking for individuals who exhibited essential CHW skills and competencies. It was also crucial that the cohort reflect diverse LGBTQIA+ and intersectional identities. Figure 1 displays a word cloud of labels contributed by participants to describe their sexualities and gender identities. Table 2 shows basic demographic information describing the cohort.

**Table 2:** Participant demographics.

**Races/ethnicities**	**Black**	**5**
	White	3
	Latinx/Latine	2
	Multiracial	1
Education levels	Bachelor's degree	5
	Some college credit	5
	Associate's degree	1
	Master's degree	1

In July 2021, the cohort gave feedback on the training *via* a half-day virtual theater testing session of two sample units. Theater testing is a methodology where individuals demonstrate the content of a program to a relevant audience to elicit feedback and opportunities for improvement (39). Specifically, two team members acted as training facilitators and presented 2 h worth of content to the cohort as if they were engaged in the specialty track training. After the facilitators presented the sample units, the cohort provided qualitative, large-group feedback. This feedback focused on both units and the entire curriculum, including topics and subtopics covered, and learning objectives. Based on the feedback, the team revised the curriculum and materials. After concluding core competency training, the cohort took the 30-h training in December 2021. The cohort provided general quantitative feedback on both trainings using the standard, Likert-item evaluative survey given by CCHA to all training participants. In addition, the cohort provided qualitative feedback about both trainings in two focus groups, comprised of 5–6 people each, which occurred in March-April 2022. Table 3 displays sample items from each of the feedback instruments.

**Table 3:** Feedback instrument sample items.

**Feedback instrument**	**Sample items**
Theater testing focus group	• How well did today's presentation describe the health issues, questions, and concerns experienced by you and your communities? How about the curriculum?
	• If you could change one thing about today's presentation, what would it be? How about the curriculum?
CCHA evaluation survey	• I am confident in my ability to use culturally appropriate communication skills when serving the community (Likert scale)
	• I am able to accurately describe the community and population I serve. This includes an understanding of their major health issues, social determinants of health, and disparities (Likert scale)
Post training focus groups	• If you had a friend or colleague interested in attending this training, what would you tell them?
	• Having completed this training, what other areas or topics would you like additional guidance and support on?

This paper focuses on specific feedback from the focus groups after the training. The research team qualitatively analyzed the feedback for themes using thematic analysis (40). The team imported the verbatim focus group transcripts into NVivo, a qualitative research analysis environment. Three team members then coded 20% of the data (transcript excerpt) line-by-line using open coding (41). The team members then met to review initial codes, combining and condensing them based on this discussion. The team resolved disagreements using NVivo's coding comparison query, which identifies coding discrepancies that served as a starting point for conversation. The team then applied the initial codes they decided on to the remainder of the transcripts and met again to identify themes or larger patterns describing what participants were saying. The team then created a codebook with themes and their related codes. Each code had a definition followed by an illustrated transcript excerpt. Since constructivist grounded theory traditions informed analysis, the team deemed inter-coder reliability calculations inappropriate due to the analytic process's iterative, recursive nature (42). To protect confidentiality, the team assigned cohort members a random number.

### 5.2. Themes from focus groups

#### 5.2.1. Representation of LGBTQIA+ experiences, identities, and issues in training content

The cohort noted the importance of revising the specialty training to balance content between those who might take the training with backgrounds in LGBTQIA+ identities and issues and those without these backgrounds. Participant 757 stated that parts of the training were “redundant” given the cohort's lived experiences and history of community health outreach, engagement, and support. However, the same participant cautioned that “not everything's gonna maybe sound redundant to [other cohorts]. And I have to constantly remind myself that [future cohorts] are people that never heard of this.” Participant 265 stated: “If you're gonna be working with the queer community, and you're not already a really knowledgeable member, [this training] is supposed to help you do that.”

While a team of many members with LGBTQIA+ identities developed and facilitated the training, not all identified as LGBTQIA+. Both focus groups expressed the perception that specific facilitators without LGBTQIA+ identities may have sometimes felt discomfort discussing LGBTQIA+ identities and issues. Participant 251 stated, “I don't think there's a level of comfort there yet,” further identifying “moments of awkwardness that I think we felt, and it was like, oh, are you having a, well, this is hard for you.”

#### 5.2.2. Application of training to CHW practice and provider education

The training informed cohort members' practices as CHWs. Participant 251 reported that the training made him more proactively and intentionally think about opportunities to engage in client-centered advocacy: “I've actually been listening more in various places that I go. So even my own doctor's appointments, um, things like that, like I'm listening more and paying attention to things that would make folks uncomfortable.” Participant 757 addressed how the training informed her professional practice and the importance of health worker education by describing plans for delivering the training to Spanish-speaking CHWs: “I'm gonna co-facilitate [the training] for people at [organization] and do it in Spanish. That would be very helpful, very helpful because it has a bunch of stuff that are everyday questions from my coworkers.”

Participant 757 highlighted provider education as a form of advocacy, stating that the purpose of “creating and troubleshooting” the training is to “teach others how to, I think providers, I think, I mean, this should be something across the board, especially here.” The participant noted that the specialization must be adapted for medical, nursing, and professional development contexts. Since the full training might be “too long, or because it's too many hours,” the participant suggested compiling essential training content into “a cheat sheet where you can like actually bring somebody for like an hour [who] can do like a lunch and learn.” The participant noted that the training should be extended to all staff interacting with LGBTQIA+ clients: “This needs to be offered to not only a healthcare provider but the reception upfront.”

#### 5.2.3. Accommodating different learning styles and learners in training delivery

This theme and the following address feedback on the core competency and specialty training. The cohort identified several elements of the learning environment and structure that could be improved. Participant 251 suggested considering additional learning styles when designing and organizing training content: “I'm a visual learner. And so, like even having a color coding, you know, that I've got, okay, these things need to go for here.” Participant 265 noted that supplemental learning materials, such as “websites, videos, questionnaires” for each topic, would “allow us to get deeper into the topic we're working with.” Participant 251 expressed “stress” and “anxiety” that emerged due to training demands.

Participants also shared feedback specific to each training. Participant 251 suggested creating a “study guide for the specialty training.” Participants expressed negative affective feelings about the core competency training centered on the CHW certification exam. Participant 265 stated that the cohort was “freaking out” about the exam, while Participant 35 described the exam as “nerve-wracking.”

#### 5.2.4. Accessing formal and experiential authority and expertise

Participants identified the participatory learning elements of both trainings as valuable in strengthening their connections to others with experiential authority and expertise. Participant 35 said that before the training, they felt like the “lone” LGBTQIA+ CHW in their specific region. By engaging with other cohort members during participatory learning activities, the participant discovered their cohort mates were “doing and thinking and asking the questions, just like I'm doing and thinking and asking the questions,” which facilitated “getting to build bigger partnerships” and “access to [interpersonal] resources.”

The cohort also identified the certification process as critical to advancing their legitimacy as CHWs. Participant 251 explained: “We live in a society where having that certification leads credibility to what we do,” despite the fact “that we already do a whole lot of these things … having that official certification, someone is like more likely to listen.” One crucial implication of certification was the potential to connect with new job opportunities. Participant 757 described cohort members expressing a desire to “work with [CHW organizations]” and asking, “do they need community health workers? I'm certified now.”

## 6. Discussion

### 6.1. Lessons learned

Cohort feedback and facilitator experiences denote several lessons. One relates to the need in the CHW field for this type of training. As cohort members shared, the training is particularly relevant for non-LGBTQIA+ CHWs with limited experience in LGBTQIA+ identities and issues. While some content may have been repetitive for the cohort, they said that the training increased their capacity for community-based advocacy, suggesting the training's relevance for CHWs who belong to LGBTQIA+ communities.

Content from the training is also extensible to other healthcare contexts like medicine and nursing. Of course, facilitators may need to adapt the methodology and instructor choice to their pedagogical style, such as a CHW and nurse co-facilitating training for nurses. Based on prior research surveying medical provider knowledge of LGBTQIA+ experiences, identities, and needs, training content would need to focus on increasing informational knowledge and addressing provider biases. For this reason, modules covering the terminology and history of LGBTQIA+ identities, resources and strategies for LGBTQIA+ health promotion, and advocacy and outreach to LGBTQIA+ people and communities would be particularly relevant. Additionally, content from the LGBTQIA+ health issues module should be integrated into the training, primarily centered on issues that practitioners have less knowledge about, such as gender-affirming care (43).

Feedback affirmed the importance of CHWs and members of LGBTQIA+ communities co-creating and facilitating course content. This strategy ensures that facilitators have enough knowledge about LGBTQIA+ identities and issues to exercise flexibility during facilitation based on the lived experiences and prior knowledge of training participants. Some groups may be new to these topics and require basic entries. Others, like the cohort, may be intimately familiar with these topics and appreciate a deep dive into underrepresented ones like polyamory and kink. A related issue entails addressing the biases or even outright discrimination that certain CHWs taking the training may have toward LGBTQIA+ identities and certain identity intersections (e.g., a queer Black man). While queer pedagogical principles informing the course content give training participants multiple opportunities to identify and challenge their biases, there may be other situations where participants are recalcitrant to incorporating new ways of knowing into their practice. In these instances, there must be careful consideration made by the training team of how to moderate participation in training. One potential avenue may be a brief questionnaire that prospective training participants complete before the training that attempts to gauge their receptivity to unlearning homo- and transphobia. Another idea would be an exercise around positionality during which all training members analyze and share their lenses, thereby helping the cohort start from a place of understanding that their experiences and perspectives will present strengths and challenges to their experiences during the course.

Additional insights suggest reinforcing that cultural humility is a lifelong process during training. Facilitators and participants have much to learn from each other. Engagement guidelines like the “oops and ouch” method are informative here. Facilitators encourage individuals to say “ouch” when someone says something that hurts. In return, the person who said the hurtful thing is encouraged to say “oops” and apologize for how their intentions did not match their impact. The person then would be encouraged to do additional research to understand why this mismatch occurred. With this ground rule established for constructive and respectful dialogue, learners can become aware of their biases, microaggressions, and prejudices. As a facilitator, encouraging and participating in this practice can increase participant trust, which might make them more willing to openly address perceptions of facilitator discomfort in discussing certain LGBTQIA+ topics.

A final lesson relates to both trainings, as cohort members identified the need to enhance their adult learning elements. As evidenced in Section 5.2.3, cohort members expressed stress and anxiety with aspects of the training. Such expression of negative affect likely represents the cohort's background as adult learners who have not necessarily been in educational settings or taken an exam for a long time. During the training, the team designated a contact from CCHA who was not involved in the trainings to be available to the cohort for them to express concerns. That contact would then communicate these concerns back to the team. In actuality, the cohort tended to communicate with the research team members about their concerns, who then shared them with the training team. The training team addressed the concerns reactively, such as by creating a study guide for the certification exam. Future iterations of the training can proactively consider these adult learning concerns by engaging in strategies like creating an online learning platform with scaffolding for course requirements and CHW credentialing procedures and providing secondary course materials. If available, an instructional designer may consult on this platform's development. Facilitators can also integrate scaffolding into the beginning of each lesson by quickly reviewing the course content, how to access it, and how learners can use it. Finally, all training materials should be reviewed for accessibility, such as through a program like Quality Matters (https://www.qualitymatters.org).

### 6.2. Practical implications

Implications from the specialty training's design, testing, and feedback related to how it can be improved and iterated for different audiences and contexts. The training team is incorporating cohort feedback into the curriculum to offer a revised version to CHWs throughout the state and beyond. These changes will be iterative and continue as the training is taught since information and terminologies are constantly changing. The team is also integrating cohort members' lived experiences as case studies during facilitation.

Further, the team is open to exploring adapting the training to different contexts, both within and outside CHW professional development. An example of a potential adaptation is to shorten the training into a half-day workshop for CHWs, medical and nursing professionals, and staff. This adaptation would also address healthcare workers' noted lack of education and professional development on LGBTQIA+ experiences, identities, and issues (27). Connecting nursing and medical professionals and staff to CHWs facilitating this training opens new avenues for potential partnerships.

### 6.3. Role of CHW cohort in co-creation

A final point of discussion reflects on the role of the 11 LGBTQIA+ CHWs in co-creating the curriculum. The team noticed that this feedback became more detailed over time as the cohort became more familiar and comfortable working with the team across the 2-year Project. It also presumably helped that many team members identified as LGBTQIA+, which established a shared experiential understanding of this population's larger health challenges. Two cohort members joined the CCHA training team working on specialty training revisions. This situation has ensured that revisions attend to cohort feedback since they are made by individuals who still communicate with the other cohort members. Further, these members might be more comfortable disclosing specific feedback to their cohort members.

These observations suggest that others who may wish to engage in similar work should adopt strategies for engendering long-term, sustainable relationships with the communities for whom the training is directed. Not doing the work and giving resources toward establishing these relationships can potentially lead to more surface-level feedback not reflective of what the community wants.

## 7. Limitations

Project limitations related to the learning environment and feedback received. The learning environment was shaped by SC CHW Credentialing Council rules that required all participants always to keep their cameras on. However, this requirement could constitute an invasion of privacy if participants are in private spaces where they do not wish for a camera to intrude. In some cases, having the camera on was a safety concern, as participants would stream parts of the training from their phones while driving. While the facilitators encouraged these participants to refrain from this activity, the credentialing requirements may have pressured participants to do so to receive training credit.

Video streaming requires stable, consistent access to technology, which was not a condition shared by all participants. It was common for participants with unstable access to be kicked out of the Zoom platform, causing them frustration. Ableist assumptions pervaded the learning environment, such as a fast delivery pace and lack of readily available accommodations for different learning styles (e.g., lack of closed captioning in some videos).

Due to scheduling difficulties, two cohort members did not participate in the focus groups following the training. While cohort feedback is invaluable and reflects a rich diversity of identities and experiences, cohort members are not community spokespeople. Therefore, facilitators must continue iterating and testing the specialty training with new cohorts.

A final constraint is balancing the need for specialty training and its magnitude with time constraints. While cohort feedback was predominately additive, subsequent revisions must balance these additions within a 30-h constraint. A way to address this concern is by facilitators creating broad learning objectives for the full training (rather than objectives at each level, which is how the training is currently designed) and tailoring content to address these objectives.

## 8. Conclusion

The development of a specialized training course for CHWs about how to reach, better understand, and serve members of the LGBTQIA+ population in culturally appropriate and humble ways helps fill a gap in the CHW field and is a critical step in advancing CHWs' ability to work with diverse individuals in their communities. Incorporating this training into CHW education and skill development processes can enhance access to healthcare and other health and social resources that LGBTQIA+ individuals may benefit from. The methods used to develop the curriculum are notable, as it was co-created by CHWs, community-engaged researchers, and leaders within LGBTQIA+ populations, illustrating a best practice in the CHW and community engagement fields. The intentionality around incorporating popular education, queer pedagogy, and cultural humility was both purposeful and essential. In the future, the training and curriculum development teams will continue to incorporate feedback and lessons learned from the initial cohort and research process to revise and shape a training program that can benefit CHWs and other professionals and enhance services for LGBTQIA+ communities.

## Data availability statement

The original contributions presented in the study are included in the article/supplementary material, further inquiries can be directed to the corresponding author.

## Ethics statement

The studies involving human participants were reviewed and approved by University of South Carolina Institutional Review Board. The patients/participants provided their written informed consent to participate in this study. Written informed consent was not obtained from the individual(s) for the publication of any potentially identifiable images or data included in this article. No potentially identifiable human images or data are presented in the manuscript.

## Author contributions

The authors confirm contribution to the paper as follows: Project conceptualization and design: VK and JS. Development of training materials and facilitation of training: VK, CB, MG, and SC-K. Data collection: VK and MG. Analysis and interpretation of results: VK. Draft manuscript preparation: VK, JS, CB, MG, and SC-K. All authors reviewed the results and approved the final version of the manuscript.
